# What happens at night? Differentiating within-day and overnight affective inertia

**DOI:** 10.1080/02699931.2025.2603460

**Published:** 2025-12-26

**Authors:** Anna J. Lücke, Stacey B. Scott, Martin J. Sliwinski, Joshua M. Smyth, Wolfgang Viechtbauer, Andreas B. Neubauer

**Affiliations:** a Institute of Psychology, RWTH Aachen University, Aachen, Germany; b Department of Psychology, Stony Brook University, Stony Brook, NY, USA; c Center for Healthy Aging, Pennsylvania State University, University Park, PA, USA; d Department of Psychology, Ohio State University, Columbus, OH, USA; e Department of Psychiatry and Neuropsychology, Maastricht University, Maastricht, The Netherlands

**Keywords:** Affective inertia, intensive longitudinal data, overnight intervals, experience sampling, ambulatory assessment

## Abstract

Affective inertia – the degree to which affect persists over time – has, for example, been linked with neuroticism, depressive symptoms, and increased distress. The typical statistical approaches modelling affective inertia as autoregression largely ignore that assessment periods covering several days also include overnight intervals which may bias affective inertia. In this study, we thus aimed to test (1) whether affective inertia differs within-day and overnight and (2) whether within-day and overnight inertia are differentially associated with psychological functioning (e.g. personality, perseverative thoughts, stress). We operationalised within-day inertia as the autoregression of affect from one timepoint to the next during the same day and overnight inertia as the autoregression of affect from the previous night to the next morning. We used data from the ESCAPE project including 254 ethnically and economically diverse participants (25–65 years) who participated in up to three 14-day measurement bursts with five daily beeps. We found significant within-day and overnight affective inertia in positive and negative affect. Overnight inertia substantially exceeded within-day inertia that would be expected for the longer overnight interval, indicating affective inertia differs within-day and overnight. This research highlights the importance to disentangle within-day and overnight intervals when studying affective inertia.

Affective inertia is the degree to which affect persists over time. While some degree of inertia is likely useful and adaptive, strong inertia is considered a marker for emotional maladjustment (Koval et al., 2021). It has been associated with the personality trait neuroticism, negative emotionality, depressive symptoms, and increased distress and might overall represent a transdiagnostic vulnerability factor for psychopathology (Houben et al., 2015; Koval et al., 2021).

Affective inertia is typically modelled as autoregression, i.e. the degree to which one’s current affect can be explained by one’s previous affect, which requires a substantial number of repeated assessments from the same person. This type of data can be collected in laboratory studies (e.g. Koval, Sütterlin, & Kuppens, 2015), yet many studies employ the experience sampling method (ESM), ecological momentary assessments (EMA), and daily diary designs to capture intensive longitudinal data (e.g. Bagnara et al., 2024; Rowland et al., 2020; Sperry et al., 2020). These types of ambulatory assessments allow assessing information repeatedly, in real-time, in people’s daily lives.

The statistical models usually applied in this context (e.g. multilevel models, dynamic structural equation models; Hamaker et al., 2018; Sperry et al., 2020) all assume one constant process where the influence of a previous affective state decreases with increasing time lag. Some models, such as dynamic structural equation models DSEM or continuous time models, explicitly include the possibility to account for heterogeneous time lags, which are likely the norm rather than the exception in most applications. Nevertheless, these models still assume that affective inertia decreases monotonically and exponentially with longer measurement intervals. By default, the same autoregressive process is assumed to apply to intervals within days and across nights. However, day and night are associated with different states of consciousness which may differently impact affective inertia. Thus, it might not be reasonable to assume that the same process governs affective inertia during the day and overnight. To account for this, another frequently used approach in the literature on affective inertia is to exclude overnight intervals altogether by replacing the first or last assessment of the day with missing values (e.g. Rowland et al., 2020; Sperry et al., 2020). This approach implicitly makes the rather strong assumption that there is no affective inertia overnight, which may also not be realistic. In this study, we therefore aim to separately model *within-day* and *overnight affective inertia*. Furthermore, we will analyse whether within-day and overnight affective inertia differ and may be differentially associated with aspects of psychological functioning. If this is the case, this would provide further support for the supposition that within-day and overnight affective inertia might be functionally different processes.

## Affective inertia

Affective inertia has been linked with several psychological constructs. Research has mainly focused on inertia in negative affect (NA inertia). Inertia in positive affect (PA inertia) has also been considered, although its links to well-being and psychological functioning have been less consistent in previous research (Houben et al., 2015; Koval, Sütterlin, & Kuppens, 2015; Wenzel & Brose, 2023).

Different personality traits have been linked with affective inertia. Despite somewhat mixed evidence, neuroticism stands out as one of the traits most consistently linked with increased NA inertia (e.g. Suls & Martin, 2005) as well as PA inertia (e.g. Koval, Sütterlin, & Kuppens, 2015). Along the same lines, stronger trait rumination has been linked with stronger NA inertia from day to day (Brose et al., 2015) and in more closely spaced momentary assessment data (Koval et al., 2012). Links of affective inertia with different forms of psychopathology have also been reported, most notably with depression and depressive symptoms (e.g. Brose et al., 2015; Hamaker et al., 2018; Nelson et al., 2020). However, these associations have also not been consistently observed (e.g. Dejonckheere et al., 2019; Sperry et al., 2020; Thompson et al., 2021). Taken together, previous findings largely suggest associations of stronger affective inertia with higher levels of negative functioning indicators and lower levels of positive functioning indicators, emphasising that stronger affective inertia (especially NA inertia) is an important indicator for emotional maladjustment (Koval et al., 2021).

While the exact mechanisms driving links of affective inertia with emotional (mal)adjustment are currently unknown, (too) high inertia is often interpreted as inflexibility, where affect does not adaptively respond to situational demands or regulative efforts (Koval & Kuppens, 2024). However, these adaptive processes are crucial for effective emotional functioning. Empirical findings and theoretical considerations suggest that there are likely internal processes (e.g. emotion regulation, reduced recovery; Bagnara et al., 2024; Koval, Sütterlin, & Kuppens, 2015) and external factors (e.g. the intensity of negative events; Koval, Brose, et al., 2015) at play to link affective inertia and emotional (mal)adjustment. This suggests that affective inertia may not be one stable trait-like characteristic but could also differ with situational characteristics and emotion regulatory behaviour.

In light of this, research has recently started to see affective inertia as a dynamic process that may vary across time and situations within-persons as well (Koval & Kuppens, 2024). While one can of course not generalise from between-person associations to within-person processes, some factors that are associated with affective inertia on the between-person level lend themselves as likely predictors (and potential mechanisms) within-persons as well, for example, momentary rumination, stress processes, and sleep.

It seems intuitive that momentary rumination could underlie the observed association between habitual rumination and stronger affective inertia (Brose et al., 2015). Recent research supported this for NA inertia and to a lesser degree for PA inertia (Bagnara et al., 2024). Anticipating social stress reduced affective inertia for people more vulnerable to social stress (Koval & Kuppens, 2012). The authors interpreted this as potentially indicating short-lived success of emotion regulation attempts which then results in increased fluctuations in affect. Lastly, initial results indicated that better sleep quality may adaptively support affective well-being by reducing affective inertia on days with lower well-being and increasing affective inertia on days with higher well-being (Zhou & Fingerman, 2024). This aligns with the supposition that better sleep may be linked with better emotion regulation during the next day (Fairholme & Manber, 2015; Lücke et al., 2022).

## Affective inertia overnight

Affective inertia is typically modelled as autoregression between time-ordered assessments of affect. This is often done in the context of dynamic structural equation models (e.g. Hamaker et al., 2018), or by including the previous timepoint’s assessment as a lagged predictor in a multilevel model context (e.g. Frérart et al., 2023). Under the assumption of an autoregressive process, the size of these lagged associations depends on the time interval between assessments with associations exponentially decreasing with increasing time intervals. The typically used models thus assume that there is only one autoregressive process (which depends on time between assessments) governing affective inertia. However, it may not be reasonable to assume that the same process applies to affective inertia while people are awake and going through their daily lives and while they are asleep during the night.

## Theoretical considerations

First of all, people mostly sleep at night, which is associated with reduced consciousness and reduced input from the outside world. This alone makes it unlikely that overnight affective inertia should be the same as during the day with the only difference being the longer interval. However, based on theoretical considerations, overnight affective inertia could differ from within-day affective inertia in several different ways. If we considered sleep as a period with no active emotional processing or regulation and no outside input, this would suggest very high affective inertia during the night. In the most extreme manifestation, people should wake up with the exact same affective state they went to bed with (perfect autoregression: affect after waking up would be perfectly predicted by affect when going to sleep). However, we know from previous research that sleep can facilitate emotional processing, suggesting that sleep works as a kind of “emotional reset”: This would predict that an individual’s affect returns to their long-term equilibrium during the night (Goldstein & Walker, 2014). Based on this proposition one might thus rather expect that people’s affect in the morning should be completely independent of their affective state in the previous evening. Both of these predictions (perfect autoregression or complete reset) seem unlikely based on theoretical considerations and empirical evidence. It seems plausible that, for example, emotional processing and emotion regulation occur during the night, but possibly slowed down compared to within-days or taking a different form. That is, affective inertia may simply function differently during the night.

A recent approach has formalised and labelled these different options regarding the process of affective inertia or autoregression overnight compared to within-day: continuing, pausing, stopping, and being different at night (Berkhout et al., 2025). A continuing process would mean that affective inertia overnight is reduced to the degree implied by the longer interval between assessments overnight. A process pausing at night would predict complete inertia (i.e. perfect autoregression) with affect in the morning being completely determined by affect the previous night. A process stopping would imply no affective inertia whatsoever with affect in the morning being completely independent of affect the previous night (i.e. zero autoregression). Lastly, if none of these three processes appropriately describe what is happening, we would consider affective inertia different overnight compared to within-days.

## Empirical findings

Despite these potentially important differences, the topic of affective inertia overnight has only recently started to receive more attention in empirical research. Research has mainly either ignored the presence of overnight intervals and treated them the same as within-day intervals (e.g. Wen et al., 2022) or explicitly excluded them from analyses (e.g. Rowland et al., 2020; Sperry et al., 2020), thus assuming no inertia overnight. After some early work which did not observe overnight affective inertia (Suls et al., 1998), only three recent studies have explicitly analysed within-day and overnight affective inertia separately (Berkhout et al., 2025; Frérart et al., 2023; Minaeva et al., 2021). These studies applied very different approaches and have not come to clear conclusions.

Minaeva et al. (2021) analysed affective inertia in currently depressed, previously depressed, and never depressed participants and observed substantial within-day and overnight affective inertia across all groups. Overnight affective inertia in NA was strongest for people in the currently depressed group compared with the other groups whereas PA inertia did not differ. Longer sleep duration was associated with stronger overnight NA inertia in the never depressed group, but with weaker overnight NA inertia in both the currently and previously depressed groups. The authors conducted separate analyses for within-day and overnight affective inertia and did not assess whether these processes differ. Frérart et al. (2023) observed no significant differences between NA or PA inertia within-day and overnight and no (differential) associations with depressive symptoms or sleep quality based on two daily assessments of affect taken 12 h apart. However, with only two beeps per day assessing affect 12 h apart, the analyses were based on evenly spaced data, which is not a common design to collect intensive longitudinal data. Thus, this previous research leaves open the question whether momentary affective inertia during the day (based on more intensive ambulatory assessment data with several beeps per day) and overnight affective inertia are governed by the same process and only differ in terms of the time interval between assessments or whether daily and overnight inertia are based on separate processes. Neither of these studies considered potential differential associations with aspects of psychological functioning other than depression and sleep.

The third study by Berkhout et al. (2025) took a more methodological approach, comparing the four potential processes (continue, pause, stop, different) that might describe affective inertia overnight by applying a novel method to intensive N = 1 data (5.7 assessments per day over 141 days for one person) for several repeatedly assessed variables (e.g. negative affect, positive affect, physical symptoms). The most probable autoregressive process (continue, pause, stop, different) differed across the analysed items, suggesting that whether or not within-day and overnight inertia converge is construct-dependent. Importantly, this study included only one participant, and leaves open the question to what extent typically reported between-person differences in inertia are related to indicators of psychological functioning.

## Open questions and the current study

Overall, previous studies have not come to clear conclusions regarding differences in within-day and overnight affective inertia and have so far not examined how within-day and overnight inertia processes may be differently associated with broader aspects of functioning. In this study we thus aim to examine a broader range of (potential) covariates of within-day and overnight affective inertia by including several person level and momentary variables that may be associated with affective inertia. Concretely, we focus on neuroticism, habitual rumination, depressive symptoms, anxiety symptoms, perceived stress, and life satisfaction, all of which have previously been linked with affective inertia (e.g. Gilbert et al., 2019; Houben et al., 2015; Koval et al., 2012; Suls et al., 1998; Wang et al., 2020). On the momentary level we include perseverative cognitions, sleep quality, and stressor experiences which have also been linked with affective inertia (e.g. Bagnara et al., 2024; Koval, Brose, et al., 2015; Zhou & Fingerman, 2024). One could speculate that some of these factors may be differentially associated with within-day and overnight inertia. For example, perseverative cognitions may be more strongly associated with increased inertia within-day compared to overnight because dwelling on negative experiences requires being awake and conscious. In contrast, better sleep quality could matter more for overnight compared to within-day affective inertia as effects of sleep quality on daily emotional functioning are usually quite small (e.g. Lücke et al., 2022; Könen et al., 2016).

Overall, this study thus aims to address two main research questions.

RQ1: Do within-day and overnight affective inertia differ?

RQ2: Are within-day and overnight affective inertia differentially associated with psychological functioning?

To address these questions, we analysed data from three waves of the Effects of Stress on Cognitive Aging, Physiology and Emotion (ESCAPE) measurement burst study (Scott et al., 2015) which includes data for up to 42 days with a total of up to 210 assessments per participant. We present a simple way to model and test separate processes of autoregression within-days and overnight and to analyse their differential associations with different aspects of psychological functioning.

## Materials and methods

### Participants

The ESCAPE study recruited an age-stratified, ethnically and economically diverse sample of participants aged 25–65 years old living in the Bronx, New York. The 254 participants included in this study were on average 46.4 years old (*SD* = 11.0) at the start of the study and mainly women (65%). Most participants self-reported their race/ethnicity as Black or African American (69%), Hispanic (28%), or White (16%); multiple answers were possible. For more detailed sample characteristics see Supplementary Table S1.

The sample size was determined by the available data in the ESCAPE study which was originally powered to detect small to medium effects of perseverative cognitions on cognitive performance in the short – and long-term (Scott et al., 2015). The sample descriptives are based on people included in the analyses in the current paper. We did not exclude any participants based on person-level variables, however we excluded data from pilot participants in Wave 1 who answered only a subset of affect items, which resulted in 11 of those participants, who only participated in Wave 1, not being included in our analyses. Additionally, we excluded non-prompted data points, i.e. data that was entered outside of scheduled beeps (7%) and duplicate entries in the data frame (0.4%).

### Procedure

ESCAPE is a measurement burst study (Sliwinski, 2008) including four waves of experience sampling embedded in a longitudinal study (data collection from 2012 to 2016); for this study we used data from the first three waves which were separated by nine-month intervals.^1^ Each wave of data collection started with participants completing a baseline survey at home after which they came to the lab for cognitive tests and to receive smartphones for the experience sampling burst phase. They then answered up to 7 brief surveys per day for 14 consecutive days using the smartphones. In the morning survey, they reported their sleep and expectations for the day, in the five daily beeps they reported on recent stressful experiences, activities, emotions, and thoughts. In the evening survey they reported, e.g. their physical symptoms that day. The five daily beeps were prompted at semi-random intervals every 2–3 h (average interval = 2.6 h) by the smartphone. People were assigned to one of seven beep schedules based on their self-reported wake-time: The earliest possible beep occurred at least 55 min after someone’s habitual wake-time (i.e. earliest at 5.55 AM for people who reported waking up at 5, 6.55 AM for people who reported waking up at 6 AM etc.). The latest possible beep occurred at 11.55 PM for people who reported waking up at 11 AM. The questionnaires could be answered by participants any time after the beep occurred; the average latency was 11 min. At the end of each of these bursts, they returned for another lab visit. Participants received a reimbursement of up to 160$ per wave depending on their compliance with the protocol. In this study, we use data from the baseline surveys, morning surveys, and the five daily beeps. For more detailed information on the protocol and assessed data, please consult the study protocol (Scott et al., 2015).

The study design with five daily beeps for 14 days across three waves means that we have data for up to 210 beeps across up to 42 days per participant. There was longitudinal drop-out (Wave 1 *n* = 245; Wave 2 *n* = 178; Wave 3 *n* = 142). The compliance was around 80% for each wave which is typical for ambulatory assessment research (Wrzus & Neubauer, 2023). Our final dataset included data from 31,255 beeps, with each participant providing data for an average of 123 (*SD* = 58) beeps.

### Measures

#### EMA

##### Negative and positive affect.

At each beep, participants rated their negative affect by reporting how angry, depressed, frustrated, and unhappy they were feeling right now, and their positive affect by reporting how happy, pleased, and joyful they were feeling, and how much enjoyment they were experiencing right now on a slider scale from 0 (*not at all*) to 100 (*extremely*). The answers to these items were averaged to form composite scores of momentary negative affect and positive affect, respectively. The values were recoded to a 0–10 scale (i.e. divided by 10) to facilitate model convergence and interpretation. Composite reliability – estimated as multilevel McDonald’s ω (Lai, 2021) – was ω^w^ = 0.85 on the within-person level and ω^b^ = 0.96 on the between-person level for negative affect and ω^w^ = 0.90 and ω^b^ = 0.98 for positive affect.

##### Sleep quality.

Each morning, participants reported their sleep quality using modified items from the PROMIS sleep disturbance short form 4a (PROMIS Health Organization, 2012c). They rated their overall *sleep quality* on a scale from 0 (*very poor*) to 100 (*very good*), and whether their sleep was *refreshing*, whether they had *difficulty falling asleep*, and whether they had *trouble staying asleep* on a scale from 0 (*not at all*) to 100 (*very much*), all presented as slider scales. The latter two items were reverse-coded before calculating a daily sleep quality composite score as the average score across those four items. These values were recoded to a 0–10 scale (i.e. divided by 10) to facilitate model convergence and interpretation. Composite reliability was ω^w^ = 0.77 on the within-person level and ω^b^ = 0.86 on the between-person level.

##### Time in bed.

Each morning, participants reported the time they went to bed in the evening and the time they got up in the morning on a 12 h clock (AM/PM). We calculated their time in bed as the time difference between these two times. Because this led to several issues with unreasonably short and long times that did not match the information people reported for their total sleep time (i.e. time in bed significantly lower than reported sleep time), we recoded some values based on rules of thumb and manual checks and excluded n = 275 (3.5%) data points (see Supplement S2 for details).

##### Unconstructive repetitive thoughts.

At each beep, participants reported on unconstructive repetitive thoughts in the past five minutes. Specifically, they reported whether they were *experiencing a train of thought they could not get out of their head*, whether they had been *thinking about personal worries or problems*, and whether they were *preoccupied with thoughts of something about to happen or that might happen in the future*. They rated their agreement to each of these statements on a slider scale from 0 (*not at all*) to 100 (*very much*). We combined these ratings into an average score for momentary unconstructive repetitive thoughts. These values were recoded to a 0–10 scale (i.e. divided by 10) to facilitate model convergence and interpretation. Composite reliability was ω^w^ = 0.75 on the within-person level and ω^b^ = 0.93 on the between-person level.

#### Baseline questionnaires

##### Neuroticism.

In the baseline assessment of Wave 1, participants answered the 24-item IPIP neuroticism scale (DeYoung et al., 2016; Goldberg et al., 2006), rating how accurately statements like “I am afraid to draw attention to myself” describe them on a scale of 1 (*very inaccurate*) to 5 (*very accurate*). Reversely framed items (e.g. “I feel comfortable with myself”) were reverse-coded before averaging responses across the 24 items to obtain participants’ neuroticism score (ω = 0.88).

##### Depressive and anxiety symptoms.

In the baseline assessments of Waves 1–3, participants reported on their depressive and anxiety symptoms in the past seven days. They answered the seven items of the PROMIS depression short form 8a (PROMIS Health Organization, 2012a; e.g. “I felt hopeless”) and the seven items of the PROMIS Emotional distress anxiety short form 7a (PROMIS Health Organization, 2012b; e.g. “I felt anxious”) on a scale from 1 (*never*) to 5 (*always*). These answers were averaged to form a composite score of depressive symptoms and anxiety symptoms for each participant at each wave (ω^2l^ = 0.94 and 0.92 respectively).

##### Rumination.

In the baseline assessments of Waves 1–3, participants answered the Rumination and Reflection Questionnaire (Trapnell & Campbell, 1999). We are using the 12 items from the rumination subscale, including items such as “Long after an argument or disagreement is over with, my thoughts keep going back to what happened” which participants rated on a scale from 0 (*strongly disagree*) to 5 (*strongly agree*). The answers to these items were averaged to form a rumination score for each participant at each wave (ω^2l^ = 0.91).

##### Perceived stress.

In the baseline assessment of Waves 1–3, participants rated their perceived stress in the past month on the 14-item Perceived Stress Scale (Cohen et al., 1983). For example, they answered the question “How often have you found that you could not cope with all the things that you had to do?” on a scale from 1 (*never*) to 5 (*very often*). Composite reliability for this scale was ω^2l^ = 0.83.

##### Life satisfaction.

In the baseline assessments of Waves 1–3, participants answered the 5-item Satisfaction With Life Scale (Diener et al., 1985). They rated statements such as “In most ways, my life is close to ideal” on a scale from 1 (*strongly disagree*) to 7 (*strongly agree*). The answers to the five items were averaged to form a life satisfaction score for each participant at each wave (ω^2l^ = 0.86).

### Analytic approach

To compare within-day and overnight affective inertia we used a Bayesian multilevel modelling approach with lagged predictors. That is, we defined affective inertia as the (auto)regression of affect at time *t* on affect at *t-1*. Because previous research has shown that centreing time-lagged variables in autoregressive models can bias the within-person autoregressive slope (Hamaker & Grasman, 2014) we used the uncentered time-lagged variable to address RQ1. To separate within-day and overnight affective inertia, we created two dummy variables denoting whether the interval *t-1 – t* occurred within-day or overnight. That is, for the first measurement each morning, the dummy variable *withinday* was coded 0 and the variable *overnight* was coded 1; for all other measurements, *withinday* was coded 1 and *overnight* was coded 0.

#### Modelling overnight and within-day affective inertia separately (RQ 1)

We built separate models for NA and PA respectively.


(1) $${affect}_{dwti}=\beta_{0dwi}+\beta_{1i}{overnight}_{ti}\;\;+\beta_{2i}\left({affect}_{t-1,i}\times {withinday}_{ti}\right)\;\;+\beta_{3i}\left({affect}_{t-1,i}\times {overnight}_{ti}\right)+\epsilon_{dwti}$$

(2) $$\beta_{0dwi}=\pi_{0di}+\xi_{0dwi}$$

(3) $$\pi_{0di}=\tau_{0i}+\zeta_{0di}$$

(4) $$\tau_{0i}=\gamma_{00}+u_{0i}$$

(5) $$\beta_{1i}=\gamma_{10}+u_{1i}$$

(6) $$\beta_{2i}=\gamma_{20}+u_{2i}$$

(7) $$\beta_{3i}=\gamma_{30}+u_{3i}$$

In these models, the parameter $\beta_{0dwi}$ is the person-specific intercept. The parameter $\beta_{1i}$ shows how much someone’s affect at beep 1 in the morning differs from their average affect across daily beeps 2–5. Parameters $\beta_{2i}$ and $\beta_{3i}$ denote within-day and overnight affective inertia, respectively. As indicated by the subscript i in equation 3 and shown in equations 4–7, all of these were random parameters, allowed to vary across people $\left(\beta_{0i}-\beta_{3i}\right)$. The parameters $\gamma_{00}-\gamma_{30}$ refer to the fixed effects, the parameters $u_{0i}-u_{3i}$ refer to the random effects, i.e. the person-specific deviations from the respective fixed effects. The random intercept ($\beta_{0dwi}$) was additionally allowed to vary across study waves $\left(\xi_{0dwi}\right)$ and days $\left(\zeta_{0di}\right)$ to consider different levels of NA and PA, respectively. For further information on the rationale behind the model, see Supplement S3.

As shown in equation 3, using two dummy variables leads to easily interpretable, separate estimates for within-day and overnight affective inertia respectively. In a Bayesian context this approach further allows us to test whether the amount of affective inertia overnight with a measurement interval of $\Delta t_{night}$ (that is, the interval between t-1 and t when *overnight* = 1) is identical to the amount of affective inertia we would expect within-day for a measurement interval of $\Delta t_{night}$ (that is the interval between t-1 and t when *withinday* = 1). In Bayesian estimation, based on Markov chain Monte Carlo methods, we can use the posterior estimates of model parameters in each iteration of the model to calculate new parameters and obtain their respective posterior distributions as well.

To address RQ1, we calculated a new parameter for the difference between the expected within-day autoregression for the length of the average overnight interval as $\gamma_{30}-\gamma_{20}^{5.3}\;(b_{overnightAR}-b_{withindayAR}^{5.3})$, based on an average within-day interval $\Delta t_{withinday}=2.6\;h$ and an average overnight interval $\Delta t_{night}=13.7\;h$. To calculate the autoregression for a different time interval, we used the exponential decay function of the autoregression: The value $\gamma_{20}^{5.3}$ represents the estimated autoregressive effect of the within-day AR if two assessments were taken 13.7 h apart (i.e. 5.3 times the length of the average within-day interval of 2.6 h). Given the point estimate and 95% credible interval (CI) for this difference parameter, we can determine whether it differs from 0 – which would indicate that the AR process governing affective inertia within days does not simply continue overnight. Regarding the other possible processes for AR overnight, we can rule out a process stopping at night when $\gamma_{30}\left(b_{overnightAR}\right)>0$. To approximate whether the process may be pausing at night, we calculated a corrected overnight interval of $\Delta t_{night\;corrected}=6.0\;h$ h by subtracting the average time in bed (7.7 h) from the average overnight interval $\Delta t_{night}=13.7\;h$. This interval approximates the time awake during the overnight interval. If the process pauses at night, $\gamma_{30}(b_{overnightAR})$ would approximately equal $\gamma_{20}^{2.3}(b_{withindayAR}^{2.3})$ with $\gamma_{20}^{2.3}$ representing the estimated autoregressive effect of the within-day AR if two assessments were taken 6 h apart (i.e. 2.3 times the length of the average within-day interval of 2.6 h).

We only considered intervals between directly adjacent beeps. That means that the variable affect_t-1_ would be considered missing if people did not answer the previous beep. Intervals with missing predictors are then excluded during model estimation. To keep sample $\Delta t_{overnight}$ and $\Delta t_{withinday}$ within reasonable bounds, we only considered beeps where the time interval to the previous beep was within the interval 1–4 h (within-day) or 10–17 h (overnight) resulting in affect_t-1_ being removed in 3.1% of cases (*n* = 975 beeps) by setting this data to missing.

#### Analysing differential associations with overnight and within-day affective inertia (RQ 2)

To test whether within-day and/or overnight inertia were associated with aspects of psychological functioning (RQ2), we included each of the variables of interest in a separate model, extending the model used to address RQ1. In these models, we centred the time-lagged affect variables on their person-mean, because previous research has shown that this can lead to more precise estimation of between-person effects on the within-person affective inertia slope (Hamaker & Grasman, 2014).

Between-person variables were centred on their respective grand means, and we added their main effects as well as cross-level interactions with all other variables in the model. For model equations see Supplement S3. To separate within-person and between-person associations, time-varying within-person variables were decomposed into their within – and between-person components by person-mean centreing momentary values (i.e. subtracting each person’s mean from each momentary observation) and adding the person-mean (centred on the grand mean) as a separate predictor. Both components were then added to the model as main effects and interactions with all other variables in the model.

#### Model specification

These models were estimated with the R package brms (Bürkner, 2017) using 4 chains with 5,000 iterations each. The first 1,000 of these iterations were used as warm-up, the rest was used for parameter estimation. The models were based on standard uninformative priors. To determine convergence, we used the potential scale reduction factor (R-hat < 1.05) and parameter trace plots. The point estimates reported as results refer to the median of the posterior distribution; the 95% credible intervals are the highest density posterior intervals. Estimates with 95% CIs that do not include 0 are interpreted as significant.

## Results

Descriptive statistics for and correlations among all variables included in the models are reported in Table 1.

### Differences between within-day and overnight affective inertia

**Figure 1.**
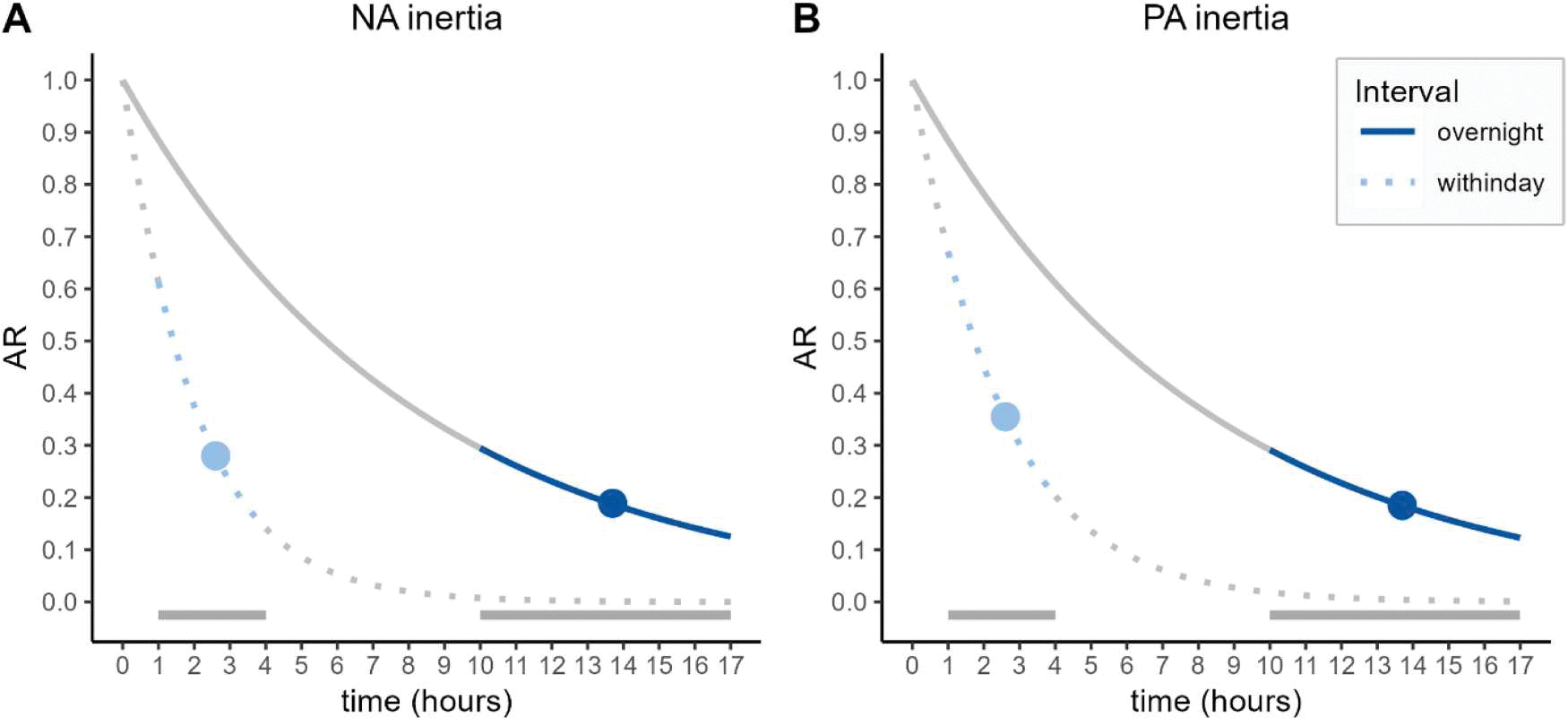
Estimated within-day and overnight autoregressive curves across time for NA (A) and PA (B). Notes: Points show the point estimates for the average intervals of 2.6 h within-day and 13.7 h overnight. Shaded parts of the curves and grey lines at the lower end of the plot indicate observed intervals within-day (1–4 h) and overnight (10–17 h). The figure illustrates that overnight affective inertia was overall weaker than within-day inertia (dark-blue point on the solid line is below light-blue point on the dotted line), but it was stronger than the inertia that would be expected if the within-day process of affective inertia continued overnight: in the range for overnight intervals (between 10 and 17 h) the dotted grey line (i.e. the implied within-day inertia) is lower than the solid line which represents the overnight inertia.

Regarding RQ1, results indicated significant within-day and overnight affective inertia in NA and PA (see Table 2 for full model results). That is, when people experienced more NA or PA this carried over to their NA and PA respectively a few hours later (within-day NA inertia = 0.280; within-day PA inertia = 0.355; see the filled light blue circles in Figure 1) as well as the next morning (overnight NA inertia = 0.189; overnight PA inertia = 0.185; see the filled dark blue circles in Figure 1). These estimates refer to the average carry-over for the average time interval between adjacent beeps (i.e. 2.6 h for within-day inertia and 13.7 h for overnight inertia).

**Figure 2.**
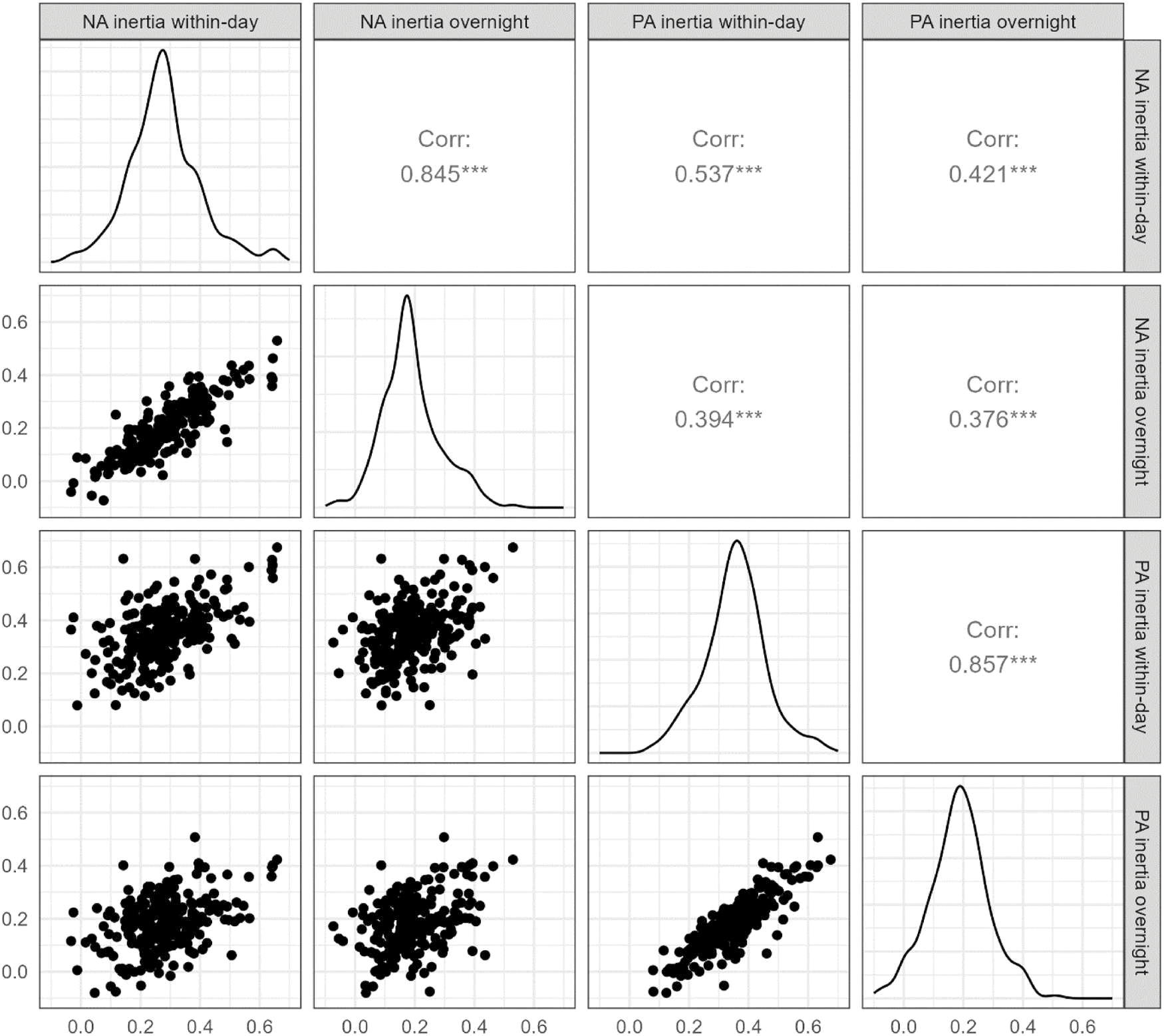
Distributions, bivariate scatterplots, and correlations of individual within-day and overnight affective inertia slopes in NA and PA.

As can be seen from random slope standard deviations (*SD within-day inertia* and *SD overnight inertia* in Table 2) and the distributions of individual estimates of affective inertia illustrated in Figure 2, people differed in how much affective inertia they experienced (both within-day and overnight). Interestingly, however, within-day and overnight affective inertia were strongly correlated – that is, people who experienced stronger within-day affective inertia also experienced stronger overnight affective inertia in the same affective domain. PA inertia and NA inertia were also correlated albeit to a lesser degree.

The significant overnight affective inertia indicates that the autoregressive process does not seem to stop overnight. Newly calculated parameters furthermore indicated that the process did not seem to continue (see overnight – withinday^5.3^) nor pause overnight (see overnight – withinday^2.3^). Thus, overnight affective inertia substantially exceeded within-day affective inertia that would be expected for intervals of Δt_night_ and Δt_night corrected_, indicating that affective inertia is most likely different overnight.

### Differential associations of within-day and overnight affective inertia with psychological functioning

Regarding RQ2, results showed that within-day and overnight affective inertia were associated with some other aspects of psychological functioning. We report the parameters for these moderation effects in Table 3; for full model results see Supplementary Tables S4–S11.

### EMA variables

When people slept better than usual, this was associated with them experiencing reduced within-day affective inertia in both NA and PA (*b* = −0.016, 95% CI [−0.022, – 0.010] and *b* = −0.017, 95% CI [−0.023, – 0.011] respectively). There is limited evidence for an association of better sleep quality with reduced overnight affective inertia in NA during that same night, with the 95% CI just including zero (b = −0.013, 95% CI [−0.026, 0.000]). Between person differences in sleep quality (i.e. better or worse average sleep quality) were not significantly associated with affective inertia (for full model results see Supplementary Table S2).

When people reported more perseverative cognitions in the past 5 min, they experienced stronger within-day inertia in NA, *b* = 0.034, 95% CI [0.030, 0.039], and PA, *b* = 0.010, 95% CI [0.005, 0.015], as well as stronger overnight inertia in NA, *b* = 0.018, 95% CI [0.08, 0.029], and PA, *b* = 0.013, 95% CI [0.002, 0.024]. The effect of perseverative cognitions on within-day NA inertia also replicated on the between-person level: People who reported more perseverative cognitions on average experienced stronger within-day NA inertia, *b* = 0.013, 95% CI [0.000, 0.026], compared to those who reported less perseverative cognitions on average. These effects were not observed for PA inertia.

A stressor occurring in people’s daily lives was associated with increased within-day NA inertia, *b* = 0.062, 95% CI [0.034, 0.089]. This means that previous affect carried over to the next measurement occasion to a stronger extent if a stressor occurred between the two measurement occasions compared to when no stressor occurred. On the between-person level, people who reported a higher proportion of situations with stressors across the duration of the study, experienced *reduced* overnight NA inertia compared to people with a lower proportion of situations with stressors, *b* = −0.269, 95% CI [−0.530, – 0.007].

#### Baseline variables

Most between-person differences were not significantly associated with affective inertia (see Table 3). People with different levels of depression, anxiety, and life satisfaction did not significantly differ in their within-day or overnight affective inertia (see Table 3), despite all of these variables largely being associated with levels of PA and NA overall as expected (see Table 1 and Supplementary Tables S5–S9).

Within-day NA inertia was stronger for participants with higher neuroticism, *b* = 0.052, 95% CI [0.009, 0.094], and higher levels of perceived stress, *b* = 0.079, 95% CI [0.042, 0.115]. Within-day PA inertia was also stronger for participants with higher levels of perceived stress, *b* = 0.072, 95% CI [0.038, 0.105]. Overnight PA inertia was stronger with higher levels of perceived stress, *b* = 0.086, 95% CI [0.027, 0.146] and trait rumination, *b* = 0.059, 95% CI [0.012, 0.104]. Full results for these models are reported in Tables S10–S12.

## Discussion

The present research examined whether within-day and overnight affective inertia converged or differed using data collected in the daily lives of an age-heterogenous sample. The results showed that people experienced affective inertia both within-days and overnight. That is, how people felt at a given moment could be partially predicted by how they felt in the past; affect carried over to the next assessment both when that past was about 2.5 h ago and when it was over 13 h ago on a previous day. Overnight affective inertia was overall weaker than within-day inertia, but it was stronger than the inertia that would be expected if the within-day process of affective inertia continued overnight. This suggests that affective inertia neither simply continues nor stops overnight, but is slowed down when people are asleep.

### Overnight differs from within-day affective inertia

Partly in contrast to previous findings, we observed significantly different within-day and overnight affective inertia (Berkhout et al., 2025; Frérart et al., 2023). These diverging findings could be explained by different study designs and modelling choices. Concretely, with 254 participants and up to 42 days per person, our study included substantially more data compared to the 127 people and 14 days included in the study by Frérart et al. (2023), which could have increased power to identify differences between within-day and overnight inertia. In terms of different autoregressive processes (continue, pause, stop, different; Berkhout et al., 2025), affective inertia being *different* overnight seemed to be the one most supported by the data in the present study. For both NA and PA, overnight affective inertia was stronger compared with within-day affective inertia extrapolated to a longer time interval, which speaks against a continuing or pausing process. The presence of a non-zero overnight inertia further speaks against a stopping process, leaving the option that the process is likely different at night compared to during the day.

Importantly, we focused on inertia in the broad categories of positive and negative affect (as is predominantly done in other research on affective inertia, e.g. Frérart et al., 2023; Kuppens et al., 2010). However, N = 1 research (Berkhout et al., 2025) considering inertia in single emotion items observed different processes for different variables. Whether our findings generalise to psychological variables beyond NA and PA is an empirical question to be assessed in future research.

We observed strong correlations of within-day and overnight affective inertia within PA and NA, respectively. That is, although the size of the autoregressive effect may differ overnight and within-day, this difference seems to be quite similar across people. NA and PA inertia were also correlated but less strongly, suggesting that they are associated but may still be governed by substantially different processes. This aligns with research showing differential associations of NA inertia and PA inertia with psychological functioning (see Houben et al., 2015; Koval et al., 2021).

### (Differential) Associations with psychological functioning

In this study, better sleep quality was associated with lower affective inertia in NA during that same night and in both NA and PA during the next day. The association with within-day inertia could indicate that better sleep may enable people to better regulate their emotions throughout the day (Fairholme & Manber, 2015; Lücke et al., 2022). Similarly, there was limited evidence that better sleep quality might be associated with reduced overnight NA inertia, partially supporting the function of good sleep as an emotional reset for negative emotions (Goldstein & Walker, 2014). Alternatively, strong overnight affective inertia might be a byproduct of high NA in the evening and the possible association of overnight inertia with worse sleep quality could thus be a result of worse sleep due to high NA when going to bed. Disentangling the cause–effect relationship between overnight inertia and sleep quality is a challenging yet important avenue for future research.

Our finding is in contrast to one previous study which did not find an association between affective inertia and sleep quality, neither within-day nor overnight (Frérart et al., 2023). However, due to the more restricted amount of data (*N* = 123 participants) compared to our study, power to find this association may have been reduced. In another study, the association of sleep with overnight affective inertia was only observed for people with past or current depression (Minaeva et al., 2021). These findings suggest that the observed associations of affective inertia with sleep quality may differ across people.

In line with previous arguments and empirical research (Bagnara et al., 2024; Suls & Martin, 2005), when people engaged in more perseverative thinking, they also experienced stronger carry-over of NA from one moment to the next within-days and overnight. This finding emphasises the potential role of emotion regulation as one mechanism driving affective inertia: with more counterproductive emotion regulation (such as perseverative thinking) NA more strongly carries over from one moment to the next and from the evening to the next morning. Given the similar finding for effects of sleep quality and known associations between perseverative thinking and sleep (Carney et al., 2010; Thomsen et al., 2003), it seems like a promising avenue for future research to consider the interplay of rumination, sleep, and overnight affective inertia in more detail.

Additionally, stronger perseverative thinking was also linked with stronger PA inertia, which could be considered somewhat surprising: Some researchers have suggested that stronger PA inertia may, in contrast to stronger NA, be an adaptive emotional dynamic, which helps to uphold positive emotional states for a longer time period (e.g. Rowland et al., 2020). However, meta-analytic results showed significant links of higher PA inertia with lower well-being; albeit to a lesser degree than for NA inertia (Houben et al., 2015), which aligns with the current findings. Future research may profit from considering the current level of affect as a potential moderator (i.e. assess whether associations are the same at high and low levels of NA and PA respectively).

Findings regarding interindividual differences in perseverative thinking seemed to depend on the assessment method. Whereas stronger average perseverative cognitions in daily life were associated with increased within-day NA inertia, stronger rumination as reported on the rumination subscale of the Rumination and Reflection scale (Trapnell & Campbell, 1999) was only significantly associated with overnight PA inertia. This is in contrast to previous research which has found trait rumination to be associated with increased NA inertia (e.g. Brose et al., 2015). These findings suggest that it may not only be rumination but also other aspects of repetitive negative thinking, such as current or future-oriented worries that could underlie stronger NA inertia.

When participants reported a stressor having occurred within the inertia interval (i.e. between t-1 and t) within-day NA inertia was increased. In the future it would be interesting to extend this research to analyse whether stressor reactivity, that is the increase in NA associated with a stressor occurring, is also associated with subsequent inertia. Previous research has considered person level averages of stressor reactivity and affective inertia and did not find significant associations (Koval et al., 2015); however, whether these two affect dynamic processes are related within-person remains an option question. The present findings further emphasise the intertwined nature of different affect dynamics such as inertia, stress reactivity, and general affect variability (Dejonckheere et al., 2019), indicating the importance of considering the joint interplay of different aspects of affect dynamics (Jahng et al., 2008).

Regarding between-person differences, we found an association of within-day NA inertia with neuroticism which aligns with previous research (e.g. Suls et al., 1998; Koval et al., 2015). Overnight affective inertia in contrast was not associated with neuroticism. It is possible that processes thought to link within-day NA inertia and neuroticism (e.g. regulation of NA) play less of a role for overnight NA inertia. However, it is also possible that the study design may have limited the possibility to find this effect; neuroticism was only assessed once at the beginning of the study and personality would be expected to change at least a little across 28 months – a change that could also have been coupled with changes in affective processes (Wrzus et al., 2021). To better capture the possibly reciprocal relation between inertia and neuroticism across time, repeated assessments of neuroticism – either trait measures separated by several months or years, or measures of personality states in EMA – would be required in future research.

We did not observe a link between affective inertia and depression in the current study. This adds to mixed findings in previous research (e.g. Sperry et al., 2020; Thompson et al., 2021; Brose et al., 2015; Hamaker et al., 2018; Nelson et al., 2020). In light of these mixed findings, it has recently been suggested that the association of NA inertia with depression may not be concurrent but lagged, i.e. affective inertia could be a risk factor for future depression rather than a marker of current depression (Koval & Kuppens, 2024). We only considered concurrent associations and thus cannot draw conclusions regarding potential lagged associations of either within-day or overnight NA inertia with depression.

We further did not observe any significant associations between affective inertia and life satisfaction. While these findings are preliminary and need to be substantiated by future research, this may suggest that affective inertia is not associated with something so broad as people’s cognitive evaluations of their life.

Stronger perceived *global* stress at the person-level (i.e. experienced overwhelm and uncontrollability dealing with life demands) was associated with increased within-day inertia in both NA and PA, which partially overlaps with previous findings (Wang et al., 2020). This finding strengthens the supposition that increased stress puts a burden on emotion regulatory processes, which was based on research showing that in phases of more stress, people also experienced stronger stress reactivity (Sliwinski, 2008). This aligns with the finding that a stressor occurring was linked with increased NA inertia. In contrast, people reporting more stressors on average, experienced decreased overnight NA inertia. Thus, it seems important to differentiate between predictors at the person-level (global stress) and the momentary level (stressor occurrence; see Hamaker, 2012) and to considering different aspects of the stress process and how they may relate to dynamics in NA and PA.

Overall, the results showed that associations of affective inertia with aspects of psychological functioning can differ both for within-day and overnight affective inertia and for different dimensions of affect (NA and PA), but no clear patterns of differential associations for within-day and overnight inertia emerged. Given the high correlation of within-day and overnight affective inertia, differential associations of these two inertias were primarily observed for time-varying variables (sleep quality and stressor occurrence). However, some variables on the between-person level were also associated only with within-day inertia (e.g. neuroticism) or overnight inertia (e.g. number of experienced stressors).

### Methodological implications for modelling affective inertia

In this study, we used a simple and easily adaptable model which allows to estimate affective inertia separately within-days and overnight, test differences therein, and assess differential associations with other variables. Our findings showed that there seem to be different processes governing affective inertia within-days and overnight which underscores the need to model them separately. However, the modelling approach we chose in this study does not consider the unequal lengths of different within-day and overnight intervals (cf., Berkhout et al., 2054). The included within-day intervals ranged from 1 to 4 h and overnight intervals from 10 to 17 h. With affective inertia as an autoregressive process being dependent on the time interval, this is not ideal. Because the considered intervals were relatively normally distributed with most of them falling within a relatively narrow range, these differences should average out in the current study. Nonetheless, it would be preferable to extend the current model or other available methods (e.g. dynamic structural equation models, Hamaker et al., 2018, or continuous time models, Driver et al., 2017) to be able to consider these different lengths of intervals while separately modelling within-day and overnight affective inertia and (differential) associations with other variables as well.

Regarding the amount of data needed to model within-day and overnight affective inertia, a simulation study conducted by Berkhout et al. (2025) indicated that in an N = 1-case about 100 days of data are needed to reliably detect whether overnight affective inertia differs from within-day affective inertia. In this study, we had data for up to 42 days per person, but data from 254 people in total and we found evidence supporting that overnight affective inertia is different from within-day affective inertia for both NA and PA. In a multilevel case, having data from multiple participants increases the amount of information available which means that fewer repeated measurements are needed to be able to identify fixed effects (Snijders, 2005). However, the multilevel approach assumes that processes are qualitatively similar across individuals. If overnight inertia is governed by different processes (stop, continue, pause, different) for different people, the chosen multilevel approach might lead to premature conclusions regarding the process for the “average person”. Future work is needed to examine both how much information is needed to identify these different processes in a multilevel case and to test the feasibility of capturing qualitative, discrete differences in the processes between people (see, e.g. Rodriguez et al., 2024).

### Limitations

Despite several strengths such as the diverse sample and many assessments per person over a long period of time, some limitations also need to be considered. First, there are some limitations regarding the applied modelling approach. As previously discussed, the models we used did not consider unequal time intervals apart from differentiating within-day and overnight intervals. With this information included, the estimated AR estimates would be more precise. Additionally, the model applied here (and in most other research on this topic) rests on the assumption, that the dynamics of affect actually follow an AR(1) process which need not necessarily be the case (e.g. Ernst et al., 2020).

Second, the data were, on average, not normally distributed but rather skewed, as is typical for ambulatory assessments of NA and PA. Although recent research concluded that this does not inflate affective inertia estimates in models such as the one used in this research, the further development of models taking non-normality of residuals into account would be advantageous for future research (Haqiqatkhah et al., 2024).

Third, this was a longitudinal measurement burst study with each participant’s assessments spanning 1.5 years but we did not consider longitudinal changes in participants “usual” level of affective inertia, that is, we did not allow participant’s inertia to vary across assessment waves. During the 1.5 years participating in this study, people may have changed in several ways, which could also impact their affective inertia. In the future, stability vs. intra-individual variability/change in autoregressive processes within-day and overnight should be empirically assessed.

Fourth, for the baseline variables rumination and life satisfaction the questionnaire instructions did not specify a timeframe; accordingly, different participants may have considered different timeframes when providing their answers.

Fifth, the results reported in this paper are based on a sample that is diverse in several aspects (e.g. racial identity, ethnicity, socio-economic status, education, age) albeit sharing a common place of residence (the Bronx, NYC). However, one recent paper including mainly East-Asian participants did not observe the well-established link between inertia and unconstructive repetitive thinking (Moeck et al., 2022). Accordingly, there might be cultural differences in processes of affective inertia that should be addressed in future research.

Last, we only considered a subset of potential covariates of affective inertia in the present work. One particular limitation is the focus on negative aspects of functioning (except for sleep quality and life satisfaction). Positive aspects of functioning that would be promising to include in future research are, e.g. mindfulness (Rowland et al., 2020), emotion regulation (Bagnara et al., 2024), or self-esteem (Koval et al., 2015) which have been linked with within-day affective inertia but have thus far not been considered in the context of overnight affective inertia.

## Conclusion

This research highlights that it is important to differentiate within-day and overnight intervals when studying affective inertia because they seem to be governed by different processes. Using a rather simple multilevel model, we showed that overnight affective inertia substantially exceeded within-day affective inertia that would be expected for the longer overnight interval. People with stronger within-day inertia also tended to experience stronger overnight inertia. However, the pattern of day-level and momentary predictors differed somewhat for within-day vs. overnight affective inertia. Future research is needed to better understand the commonalities and differences of the carryover of affective states within-days and overnight, especially on the within-person level.

## Supplementary Material

Supp 1

Supplemental data for this article can be accessed online at https://doi.org/10.1080/02699931.2025.2603460.

## Figures and Tables

**Table 1.** Descriptive Statistics and Correlations (within-person above the diagonal, between-person below the diagonal).

					Correlations												
Variable	mean	SD	iSD	ICC	NA	PA	Thoughts	Stressors	SQ	TIB	Neur	Rumin	PSS	Depr	Anx	Lifesat	Age

NA	2.21	1.54	1.32	0.52	–	**−0.573**	**0.452**	**0.378**	**−0.176**	−0.003	–	–	–	–	–	–	–
PA	6.08	1.89	1.54	0.53	**−0.463**	**–**	**−0.289**	**−0.284**	**0.205**	0.031	–	–	–	–	–	–	–
Thoughts	3.52	1.91	1.78	0.49	**0.710**	**−0.311**	**–**	**0.311**	**−0.103**	**−0.036**	–	–	–	–	–	–	–
Stressors	0.16	0.15	0.31	0.15	**0.288**	**−0.312**	**0.251**	**–**	**−0.080**	**−0.039**	–	–	–	–	–	–	–
SQ	6.38	1.76	1.62	0.47	**−0.580**	**0.512**	**−0.443**	**−0.219**	**–**	**0.116**	–	–	–	–	–	–	–
TIB	7.66	1.42	1.85	0.31	0.006	−0.003	−0.125	**−0.234**	0.031	–	–	–	–	–	–	–	–
Neur	2.49	0.63	–	–	**0.383**	**−0.370**	**0.374**	**0.213**	**−0.310**	−0.026	–	–	–	–	–	–	–
Rumin	3.01	0.78	–	–	**0.308**	**−0.324**	**0.441**	**0.207**	**−0.268**	−0.080	**0.574**	–	–	–	–	–	–
PSS	1.76	0.49	–	–	**0.431**	**−0.401**	**0.455**	**0.257**	**−0.389**	−0.072	**0.610**	**0.580**		–	–	–	–
Depr	1.93	0.80	–	–	**0.444**	**−0.347**	**0.393**	**0.291**	**−0.345**	−0.060	**0.594**	**0.552**	**0.641**	–	–	–	–
Anx	2.39	0.75	–	–	**0.429**	**−0.407**	**0.456**	**0.354**	**−0.349**	−0.051	**0.575**	**0.581**	**0.617**	**0.760**		–	–
Lifesat	3.67	1.34	–	–	**−0.300**	**0.382**	**−0.225**	**−0.173**	**0.290**	0.097	**−0.432**	**−0.407**	**−0.573**	**−0.572**	**−0.441**	–	–
Age	46.39	11.01	–	–	−0.040	**0.154**	−0.065	0.101	0.102	−0.081	−0.013	**−0.143**	−0.121	−0.016	−0.071	0.023	–
Gender	0.35	0.48	–	–	−0.050	0.088	−0.013	−0.070	0.109	−0.106	−0.108	−0.005	−0.040	−0.058	−0.088	−0.039	0.019

Notes: The column “mean” shows the average of all individual means; the column “SD” shows the standard deviation of those individual means. For the within-person correlations involving sleep quality variables were aggregated by day and person. Between-person correlations were calculated based on the individual means. **Bold** text indicates correlations with *p* < .05. NA = negative affect. PA = positive affect. Thoughts = Unconstructive repetitive thoughts. SQ = sleep quality. TIB = time in bed. Neur = neuroticism. Rumin = rumination. PSS = perceived stress scale. Depr = depressive symptoms. Anx = anxiety symptoms. Lifesat = life satisfaction.

**Table 2.** Results from Bayesian multilevel model analysing within-day and overnight affective inertia in NA and PA.

	NA		PA	
Model parameters	Estimate	95% CI	Estimate	95% CI

**Intercept**	**1.549**	1.408, 1.700	**3.962**	3.725, 4.205
**overnight**	**0.180**	0.094, 0.267	**0.785**	0.546, 1.020
**within-day inertia**	**0.280**	0.249, 0.310	**0.355**	0.326, 0.385
**overnight inertia**	**0.190**	0.154, 0.226	**0.186**	0.151, 0.222
*Calculated Parameters*				
**overnight – withinday^5.3^**	**0.189**	0.153, 0.224	**0.181**	0.148, 0.217
**overnight – withinday^2.3^**	**0.136**	0.104, 0.169	**0.093**	0.061, 0.126
*Random Effects (standard deviations)*				
**SD intercept id**	**1.049**	0.939, 1.174	**1.455**	1.287, 1.652
**SD intercept id wave**	**0.431**	0.387, 0.480	**0.409**	0.362, 0.461
**SD intercept id wave day**	**0.337**	0.287, 0.382	**0.365**	0.301, 0.421
**SD overnight**	**0.241**	0.118, 0.344	**0.735**	0.521, 0.956
**SD within-day inertia**	**0.157**	0.138, 0.179	**0.138**	0.120, 0.158
**SD overnight inertia**	**0.147**	0.116, 0.181	**0.148**	0.117, 0.181
Observations	26762		26745	
Marginal / Conditional R^2^	0.092 / 0.647		0.150 / 0.670	

**Table 3.** Model estimates for interactions of within-day and overnight affective inertia in NA and PA with aspects of psychological functioning.

	NA				PA			
	Within-day inertia		Overnight inertia		Within-day inertia		Overnight inertia	
	Estimate	95% CI	Estimate	95% CI	Estimate	95% CI	Estimate	95% CI

Within-person								
Perseverative Cognitions	**0.034**	**0.030, 0.039**	**0.018**	**0.008, 0.029**	**0.010**	**0.005, 0.015**	**0.013**	**0.002, 0.024**
Sleep Quality	**−0.016**	**−0.022, − 0.010**	**−0.013**	−0.026, 0.000	**−0.017**	**−0.023, − 0.011**	0.008	−0.004, 0.021
Stressor	**0.062**	**0.034, 0.089**	−0.004	−0.067, 0.060	−0.008	−0.037, 0.022	0.040	−0.026, 0.106
Between-person								
Perseverative Cognitions	**0.013**	**0.000, 0.026**	0.017	−0.003, 0.037	0.003	−0.010, 0.016	0.003	−0.016, 0.023
Sleep Quality	−0.000	−0.016, 0.015	−0.009	−0.034, 0.016	−0.005	−0.020, 0.010	−0.006	−0.029, 0.016
Stressor	0.021	−0.159, 0.202	**−0.269**	**−0.530, − 0.007**	−0.015	−0.186, 0.156	−0.154	−0.407, 0.097
Neuroticism	**0.052**	**0.009, 0.094**	0.011	−0.053, 0.076	0.038	−0.002, 0.078	−0.009	−0.069, 0.051
Depression	0.005	−0.015, 0.025	−0.007	−0.042, 0.028	−0.016	−0.036, 0.004	0.017	−0.016, 0.052
Anxiety	0.022	−0.001, 0.045	0.009	−0.030, 0.050	0.006	−0.016, 0.028	0.035	−0.002, 0.072
Rumination	0.017	−0.010, 0.043	0.042	−0.002, 0.087	0.022	−0.004, 0.047	**0.070**	**0.028, 0.111**
Perceived Stress	**0.079**	**0.042, 0.115**	0.025	−0.042, 0.090	**0.072**	**0.038, 0.105**	**0.086**	**0.027, 0.146**
Life satisfaction	−0.003	−0.018, 0.012	0.016	−0.009, 0.041	−0.008	−0.021, 0.006	0.002	−0.021, 0.025

Notes: **Bold** text indicates estimates for which the CI does not cover 0. For full model results please refer to Tables S2–S9 in the Supplementary Material.

## Data Availability

Data are available through the data sharing mechanism of the ESCAPE study (see https://osf.io/4ctdv/).

## Footnotes

1. We did not use data from the fourth wave because of the relatively low number of participants. We further provide analysis scripts on the OSF at https://osf.io/xkjbz/.
