# Supplementary material for: What happens at night? Differentiating within-day and overnight affective inertia
Source: Cogn Emot. Author manuscript; Available in PMC 2026 Feb 9. (PMC12884828; doi:10.1080/02699931.2025.2603460)
Supplement: Supp 1 [file NIHMS2134570-supplement-Supp_1.docx]

**Supplementary Material to**

**What happens at night? Differentiating within-day and overnight affective inertia**

Anna J. Lücke^1^, Stacey Scott^2^, Martin Sliwinski^3^, Joshua Smyth^4^, Wolfgang Viechtbauer^5^, & Andreas B. Neubauer^1^

^1^ RWTH Aachen University

^2^ Stony Brook University

^3^ Penn State

^4^ Ohio State University

^5^ Maastricht University

**Table S1**

*Sample Characteristics*

| Characteristic | n (%) |
| --- | --- |
| *Gender* |  |
| male | 88 (35%) |
| female | 166 (65%) |
| *Race/Ethnicity* |  |
| hispanic | 70 (28%) |
| white | 41 (16%) |
| black or african american | 174 (69%) |
| american indian or alaska native | 10 (3.9%) |
| asian | 1 (0.4%) |
| other | 48 (19%) |
| *Education* |  |
| grade school or less | 2 (0.8%) |
| some high school | 14 (5.5%) |
| completed high school / received GED | 46 (18%) |
| some college | 81 (32%) |
| completed college | 69 (27%) |
| graduate or professional degree | 42 (17%) |
| *Work status* |  |
| employed | 128 (51%) |
| retired | 33 (13%) |
| unemployed and looking for work | 70 (28%) |
| unemployed and not looking for work | 21 (8.3%) |
| Unknown | 2 |
| *Yearly income* |  |
| Less than 4,999 | 13 (5.2%) |
| 5,000-19,999 | 37 (15%) |
| 20,000 - 39,999 | 60 (24%) |
| 40,000 - 59,999 | 53 (21%) |
| 60,000 - 79,999 | 27 (11%) |
| 80,000 - 99,999 | 16 (6.3%) |
| 100,000 - 149,999 | 20 (7.9%) |
| 150,000 or more | 4 (1.6%) |
| no answer | 22 (8.7%) |
| Unknown | 2 |
| *Marital status* |  |
| married | 79 (31.7%) |
| divorced | 28 (11%) |
| separated | 12 (4.7%) |
| never married | 89 (35%) |
| widowed | 7 (2.8%) |
| cohabitating | 23 (9.1%) |
| other | 15 (5.9%) |
| Unknown | 1 |

**Supplement S2 – Additional information on recoding of time in bed**

Each morning, participants reported the time they went to bed in the evening and the time they got up in the morning on a 12 h clock (AM/PM). We calculated their time in bed as the time difference between these two times. Because this led to several issues with unreasonably short and long times that did not match the information people reported for their total sleep time (i.e., time in bed significantly lower than reported sleep time), we recoded some values based on rules of thumb and manual checks and excluded n = 275 (3.5%) data points. First of all, when participants reported their bed time as between 7.00 AM and 11.59 AM we assumed that they probably meant PM (*n* = 418 days, 5.3%). Similarly, when participants reported having woken up between 1.00 and 11.59 PM we assumed that they probably meant AM (*n* = 76, 1.0%). After these corrections, we flagged all cases where time in bed was more than one hour lower than the reported sleep duration, or when time in bed exceeded 12 hours (*n* = 475, 6.0%). These cases were then manually checked for plausibility taking into consideration reported sleep duration, when participants had filled out the morning and evening questionnaires, and patterns in participants usual bed and wake times. When the data provided seemed plausible in context, data was kept as is (*n* = 14, 0.2%), when corrections of AM/PM for either bed or wake times resulted in more plausible data these reports were manually corrected (*n* = 186, 2.3%), when there was no way to determine plausible times this data was set to missing (*n* = 275, 3.5%).

# Supplement S3 – Further information on the applied models

## Rationale for Model Set-Up

In the following, we explain some of the modelling choices in more detail. As described in the manuscript, we used two dummy variables to code whether a given interval *t-1– t* occurred within-day or overnight. This approach renders the model parameters directly interpretable as the autoregressive effects within-day and overnight, respectively. Alternative, statistically equivalent models with only one dummy variable would yield only one of the relevant two autoregressive effects and the difference between within-day and overnight autoregressive effects which would be less straightforward.

***Dummy main effects***

We only included a main effect for one of the two dummy variables - the variable overnight - in the model. Because we also included the intercept in the model, we could not estimate the main effects of both dummies (within-day and overnight), as this would create singularity (complete redundance) in the set of the three predictors (intercept, within-day, overnight) and make the model non-identifiable. By including the dummy overnight, the resulting regression coefficient β_1i_  yields the model predicted difference between affect at beep 1 (overnight = 1) from average affect at beeps 2 – 5 (i.e., in the reference category with overnight = 0). A positive value of β_1i_ hence indicates higher affect at beep 1 than at beeps 2-5. In an alternative, equivalent, model, we could have opted to include the dummy variable withinday instead of the overnight dummy. In that case, the regression coefficient for the dummy withinday would have yielded the model predicted difference between average affect at beeps 2 – 5 (withinday = 1) and affect at beep 1 (withinday = 0).

***Dummy interactions***

We included the interaction between the lagged affect variable with both dummies. By setting up the model in this way, the associated regression coefficients (β_2_ and β_3_; see equations below) are directly interpretable as the effect of previous affect when withinday = 1 (i.e., the within-day autoregressive effect) and the effect of previous affect when overnight = 1 (i.e., the overnight autoregressive effect). Note, however, that including both of these interactions required us to constrain the main effect of lagged affect to zero: the set of the three predictors main effect of lagged affect, the within-day x lagged affect interaction, and the overnight x lagged affect interaction are singular and these three effects cannot be estimated independently. Therefore, one of these three predictors needs to be set to zero (= removed from the regression). We chose to remove the main effect of lagged affect to directly obtain the two relevant autoregressive effects: the within-day autoregressive effect (β_2_) and the overnight autoregressive effect (β_3_).

Model Equations for Models addressing RQ2

## Baseline variables

| affect_dwti_ = β_0dwi_ + β_1i_overnight_ti_ + β_2i_(affect_pmc_t-1,i_ × withinday_ti_) + β_3i_(affect_pmc_t-1,i_ × overnight_ti_) + ϵ_dwti_ |
| --- |
| β_0dwi_​ = π_0di_ ​+ ξ_0dwi​_ |
| π_0di_​ = τ_0i_​ + ζ_0di_​ |
| τ_0i_​ = γ_00_​ + γ_01_​BP_var_i_ + u_0i_ |
| β_1i_ = γ_10_ + γ_11_​BP_var_i_ + u_1i_ |
| β_2i_ = γ_20_ + γ_21_​BP_var_i_ + u_2i_ |
| β_3i_ = γ_30_ + γ_31_​BP_var_i_ + u_3i_ |

## EMA variables

| affect_dwti_ = β_0dwi_ + β_1i_overnight_ti_ + β_2i_(affect_pmc_t-1,i_ × withinday_ti_) + β_3i_(affect_pmc_t-1,i_ × overnight_ti_) + β_4_EMA_var_pmc_t/di_ + β_5_(affect_pmc_t-1,i_ × withinday_ti_ × EMA_var_pmc_t/di_)  + β_6_(affect_t-1,i_ × overnight_ti_ × EMA_var_pmc_t/di_) + ϵ_wdti_ |
| --- |
| β_0dwi_​ = π_0di_ ​+ ξ_0dwi​_ |
| π_0di_​ = τ_0i_​ + ζ_0di_​ |
| τ_0i_​ = γ_00_​ + γ_01_​EMA_var_pm_gmc_i_ + u_0i_ |
| β_1i_ = γ_10_ + γ_11_​EMA_var_pm_gmc_i_ + u_1i_ |
| β_2i_ = γ_20_ + γ_21_​EMA_var_pm_gmc_i_ + u_2i_ |
| β_3i_ = γ_30_ + γ_31_​EMA_var_pm_gmc_i_ + u_3i_ |

pmc = person-mean centered

pm = person-mean
gmc = grand-mean centered

**Table S4**

*Model Results for Sleep Quality as a Moderator of Affective Inertia*

|  | **NA** | | **PA** | |
| --- | --- | --- | --- | --- |
| *Model Parameters* | *Estimate* | *95% CI* | *Estimate* | *95% CI* |
| **Intercept** | **2.218** | 2.060, 2.374 | **6.104** | 5.901, 6.304 |
| **overnight** | -0.011 | -0.059, 0.038 | **-0.198** | -0.278, -0.118 |
| **SQ_wpc** | **-0.042** | -0.054, -0.030 | **0.046** | 0.032, 0.059 |
| **SQ_pm_gmc** | **-0.511** | -0.598, -0.417 | **0.544** | 0.424, 0.662 |
| **within-day inertia** | **0.249** | 0.218, 0.280 | **0.312** | 0.282, 0.343 |
| **overnight inertia** | **0.142** | 0.101, 0.184 | **0.144** | 0.107, 0.182 |
| **overnight x SQ_wpc** | **-0.090** | -0.114, -0.065 | **0.151** | 0.123, 0.180 |
| **overnight x SQ_pm_gmc** | -0.002 | -0.029, 0.025 | 0.019 | -0.027, 0.065 |
| **within-day inertia x SQ_wpc** | **-0.016** | -0.022, -0.010 | **-0.017** | -0.023, -0.011 |
| **overnight inertia x SQ_wpc** | -0.013 | -0.026, 0.000 | 0.008 | -0.004, 0.021 |
| **within-day inertia x SQ_pm_gmc** | -0.000 | -0.016, 0.015 | -0.005 | -0.020, 0.010 |
| **overnight inertia x SQ_pm_gmc** | -0.009 | -0.034, 0.016 | -0.006 | -0.029, 0.016 |
| *Random Effects (standard deviations)* | | | | |
| **SD intercept id** | **1.220** | 1.108, 1.347 | **1.610** | 1.469, 1.767 |
| **SD intercept id wave** | **0.450** | 0.405, 0.501 | **0.414** | 0.366, 0.466 |
| **SD intercept id wave day** | **0.355** | 0.307, 0.397 | **0.393** | 0.337, 0.447 |
| **SD overnight** | **0.167** | 0.040, 0.240 | **0.480** | 0.409, 0.558 |
| **SD within-day inertia** | **0.160** | 0.140, 0.184 | **0.156** | 0.135, 0.179 |
| **SD overnight inertia** | **0.174** | 0.132, 0.220 | **0.148** | 0.110, 0.188 |
| Observations | 25393 | | 25377 | |
| Marginal R^2^ / Conditional R^2^ | 0.216 / 0.654 | | 0.192 / 0.679 | |

*Note.* wpc = within-person centered on the person-mean. pm = person-mean. gmc = grand-mean centered

**Table S5**

*Model Results for Perseverative Cognitions as a Moderator of Affective Inertia*

|  | **NA** | | **PA** | |
| --- | --- | --- | --- | --- |
| *Model Parameters* | *Estimate* | *95% CI* | *Estimate* | *95% CI* |
| **Intercept** | **2.225** | 2.095, 2.360 | **6.093** | 5.876, 6.319 |
| **overnight** | -0.023 | -0.067, 0.020 | **-0.169** | -0.249, -0.090 |
| **Cogn_wpc** | **0.300** | 0.291, 0.309 | **-0.227** | -0.238, -0.217 |
| **COGN_pm_gmc** | **0.576** | 0.506, 0.646 | **-0.317** | -0.433, -0.198 |
| **within-day inertia** | **0.180** | 0.155, 0.206 | **0.278** | 0.250, 0.306 |
| **overnight inertia** | **0.129** | 0.093, 0.167 | **0.147** | 0.110, 0.185 |
| **overnight x COGN_wpc** | -0.008 | -0.028, 0.012 | **0.037** | 0.013, 0.061 |
| **overnight x COGN_pm_gmc** | -0.000 | -0.023, 0.022 | **0.045** | 0.004, 0.086 |
| **within-day inertia x COGN_wpc** | **0.034** | 0.030, 0.039 | **0.010** | 0.005, 0.015 |
| **overnight inertia x COGN_wpc** | **0.018** | 0.008, 0.029 | **0.013** | 0.002, 0.024 |
| **within-day inertia x COGN_pm_gmc** | **0.013** | 0.000, 0.026 | 0.003 | -0.010, 0.016 |
| **overnight inertia x COGN_pm_gmc** | 0.017 | -0.003, 0.037 | 0.003 | -0.016, 0.023 |
| *Random Effects (standard deviations)* | | | | |
| **SD intercept id** | **1.021** | 0.926, 1.129 | **1.774** | 1.619, 1.949 |
| **SD intercept id wave** | **0.399** | 0.358, 0.444 | **0.419** | 0.373, 0.469 |
| **SD intercept id wave day** | **0.314** | 0.276, 0.350 | **0.401** | 0.352, 0.448 |
| **SD overnight** | **0.159** | 0.075, 0.220 | **0.497** | 0.429, 0.570 |
| **SD within-day inertia** | **0.137** | 0.118, 0.157 | **0.148** | 0.128, 0.169 |
| **SD overnight inertia** | **0.163** | 0.126, 0.204 | **0.154** | 0.119, 0.192 |
| Observations | 26639 | | 26630 | |
| Marginal R^2^ / Conditional R^2^ | 0.404 / 0.710 | | 0.132 / 0.699 | |

*Note.* wpc = within-person centered on the person-mean. pm = person-mean. gmc = grand-mean centered

**Table S6**

*Model Results for Stressor Occurrence as a Moderator of Affective Inertia*

|  | **NA** | | **PA** | |
| --- | --- | --- | --- | --- |
| *Model Parameters* | *Estimate* | *95% CI* | *Estimate* | *95% CI* |
| **Intercept** | **2.205** | 2.022, 2.392 | **6.105** | 5.881, 6.330 |
| **overnight** | 0.005 | -0.040, 0.050 | **-0.200** | -0.281, -0.117 |
| **Stressor_wpc** | **1.469** | 1.420, 1.518 | **-1.315** | -1.373, -1.257 |
| **Stressor_pm_gmc** | **2.941** | 1.706, 4.194 | **-4.016** | -5.565, -2.450 |
| **within-day inertia** | **0.201** | 0.173, 0.230 | **0.290** | 0.262, 0.319 |
| **overnight inertia** | **0.159** | 0.119, 0.198 | **0.154** | 0.117, 0.192 |
| **overnight x Stressor_wpc** | -0.023 | -0.140, 0.090 | **0.153** | 0.017, 0.288 |
| **overnight x Stressor_pm_gmc** | 0.083 | -0.231, 0.399 | -0.106 | -0.687, 0.465 |
| **within-day inertia x Stressor_wpc** | **0.062** | 0.034, 0.089 | -0.008 | -0.037, 0.022 |
| **overnight inertia x Stressor_wpc** | -0.004 | -0.067, 0.060 | 0.040 | -0.026, 0.106 |
| **within-day inertia x Stressor_pm_gmc** | 0.022 | -0.159, 0.202 | -0.015 | -0.186, 0.156 |
| **overnight inertia x Stressor_pm_gmc** | **-0.269** | -0.530, -0.007 | -0.154 | -0.407, 0.097 |
| *Random Effects (standard deviations)* | | | | |
| **SD intercept id** | **1.447** | 1.320, 1.590 | **1.78** | 1.628, 1.957 |
| **SD intercept id wave** | **0.448** | 0.404, 0.496 | **0.423** | 0.377, 0.474 |
| **SD intercept id wave day** | **0.358** | 0.320, 0.394 | **0.399** | 0.350, 0.446 |
| **SD overnight** | **0.180** | 0.109, 0.238 | **0.515** | 0.448, 0.592 |
| **SD within-day inertia** | **0.156** | 0.136, 0.178 | **0.146** | 0.127, 0.168 |
| **SD overnight inertia** | **0.182** | 0.147, 0.223 | **0.158** | 0.123, 0.198 |
| Observations | 26597 | | 26588 | |
| Marginal R^2^ / Conditional R^2^ | 0.129 / 0.704 | | 0.127 / 0.703 | |

*Note.* wpc = within-person centered on the person-mean. pm = person-mean. gmc = grand-mean centered

**Table S7**

*Model Results for Depression as a Moderator of Affective Inertia*

|  | **NA** | | **PA** | |
| --- | --- | --- | --- | --- |
| *Model Parameters* | *Estimate* | *95% CI* | *Estimate* | *95% CI* |
| **Intercept** | **2.207** | 2.026, 2.389 | **6.121** | 5.884, 6.352 |
| **overnight** | -0.004 | -0.052, 0.044 | **-0.198** | -0.279, -0.116 |
| **Depression** | **0.235** | 0.143, 0.326 | **-0.139** | -0.232, -0.048 |
| **within-day inertia** | **0.244** | 0.213, 0.275 | **0.311** | 0.282, 0.341 |
| **overnight inertia** | **0.165** | 0.123, 0.208 | **0.160** | 0.122, 0.199 |
| **overnight x Depression** | -0.006 | -0.057, 0.048 | 0.060 | -0.012, 0.132 |
| **within-day inertia x Depression** | 0.005 | -0.015, 0.025 | -0.016 | -0.036, 0.004 |
| **overnight inertia x Depression** | -0.007 | -0.042, 0.028 | 0.017 | -0.016, 0.052 |
| *Random Effects (standard deviations)* | | | | |
| **SD intercept id** | **1.434** | 1.308, 1.580 | **1.838** | 1.675, 2.021 |
| **SD intercept id wave** | **0.452** | 0.405, 0.502 | **0.430** | 0.381, 0.482 |
| **SD intercept id wave day** | **0.379** | 0.334, 0.419 | **0.417** | 0.363, 0.466 |
| **SD overnight** | **0.188** | 0.110, 0.253 | **0.488** | 0.419, 0.565 |
| **SD within-day inertia** | **0.164** | 0.144, 0.187 | **0.152** | 0.133, 0.176 |
| **SD overnight inertia** | **0.187** | 0.145, 0.232 | **0.161** | 0.124, 0.202 |
| Observations | 26685 | | 26668 | |
| Marginal R^2^ / Conditional R^2^ | 0.041 / 0.654 | | 0.045 / 0.676 | |

**Table S8**

*Model Results for Anxiety as a Moderator of Affective Inertia*

|  | **NA** | | **PA** | |
| --- | --- | --- | --- | --- |
| *Model Parameters* | *Estimate* | *95% CI* | *Estimate* | *95% CI* |
| **Intercept** | **2.203** | 2.025, 2.387 | **6.117** | 5.891, 6.344 |
| **overnight** | -0.004 | -0.051, 0.044 | **-0.198** | -0.278, -0.119 |
| **Anxiety** | **0.322** | 0.223, 0.425 | **-0.271** | -0.375, -0.170 |
| **within-day inertia** | **0.241** | 0.211, 0.271 | **0.310** | 0.280, 0.340 |
| **overnight inertia** | **0.164** | 0.122, 0.205 | **0.157** | 0.119, 0.195 |
| **overnight x Anxiety** | -0.034 | -0.090, 0.023 | **0.103** | 0.020, 0.185 |
| **within-day inertia x Anxiety** | 0.022 | -0.001, 0.045 | 0.006 | -0.016, 0.028 |
| **overnight inertia x Anxiety** | 0.009 | -0.030, 0.050 | 0.035 | -0.002, 0.072 |
| *Random Effects (standard deviations)* | | | | |
| **SD intercept id** | **1.419** | 1.293, 1.566 | **1.800** | 1.639, 1.977 |
| **SD intercept id wave** | **0.443** | 0.398, 0.494 | **0.421** | 0.375, 0.474 |
| **SD intercept id wave day** | **0.380** | 0.337, 0.421 | **0.421** | 0.368, 0.469 |
| **SD overnight** | **0.185** | 0.095, 0.250 | **0.485** | 0.417, 0.562 |
| **SD within-day inertia** | **0.163** | 0.143, 0.187 | **0.152** | 0.132, 0.175 |
| **SD overnight inertia** | **0.184** | 0.143, 0.230 | **0.163** | 0.127, 0.204 |
| Observations | 26685 | | 26668 | |
| Marginal R^2^ / Conditional R^2^ | 0.049 / 0.654 | | 0.052 / 0.677 | |

**Table S9**

*Model Results for Life Satisfaction as a Moderator of Affective Inertia*

|  | **NA** | | **PA** | |
| --- | --- | --- | --- | --- |
| *Model Parameters* | *Estimate* | *95% CI* | *Estimate* | *95% CI* |
| **Intercept** | **2.206** | 2.016, 2.390 | **6.110** | 5.876, 6.341 |
| **overnight** | -0.005 | -0.051, 0.044 | **-0.199** | -0.280, -0.118 |
| **Life satisfaction** | **-0.117** | -0.175, -0.061 | **0.125** | 0.064, 0.184 |
| **within-day inertia** | **0.246** | 0.214, 0.276 | **0.310** | 0.280, 0.340 |
| **overnight inertia** | **0.169** | 0.127, 0.211 | **0.162** | 0.124, 0.201 |
| **overnight x Life satisfaction** | 0.002 | -0.029, 0.034 | -0.010 | -0.059, 0.037 |
| **within-day inertia x Life satisfaction** | -0.003 | -0.018, 0.012 | -0.008 | -0.021, 0.006 |
| **overnight inertia x Life satisfaction** | 0.016 | -0.009, 0.041 | 0.002 | -0.021, 0.025 |
| *Random Effects (standard deviations)* | | | | |
| **SD intercept id** | **1.468** | 1.343, 1.614 | **1.822** | 1.235, 1.590 |
| **SD intercept id wave** | **0.449** | 0.404, 0.500 | **0.425** | 0.378, 0.478 |
| **SD intercept id wave day** | **0.376** | 0.332, 0.417 | **0.419** | 0.364, 0.468 |
| **SD overnight** | **0.187** | 0.085, 0.252 | **0.492** | 0.423, 0.569 |
| **SD within-day inertia** | **0.165** | 0.144, 0.188 | **0.150** | 0.130, 0.172 |
| **SD overnight inertia** | **0.190** | 0.148, 0.236 | **0.161** | 0.124, 0.202 |
| Observations | 26685 | | 26668 | |
| Marginal R^2^ / Conditional R^2^ | 0.036 / 0.653 | | 0.049 / 0.677 | |

**Table S10**

*Model Results for Neuroticism as a Moderator of Affective Inertia*

|  | **NA** | | **PA** | |
| --- | --- | --- | --- | --- |
| *Model Parameters* | *Estimate* | *95% CI* | *Estimate* | *95% CI* |
| **Intercept** | **2.209** | 2.032, 2.387 | **6.110** | 5.898, 6.329 |
| **overnight** | -0.006 | -0.054, 0.041 | **-0.197** | -0.277, -0.117 |
| **Neuroticism** | **0.946** | 0.658, 1.223 | **-1.133** | -1.475, -0.774 |
| **within-day inertia** | **0.242** | 0.212, 0.273 | **0.308** | 0.278, 0.336 |
| **overnight inertia** | **0.166** | 0.124, 0.208 | **0.165** | 0.126, 0.203 |
| **overnight x Neuroticism** | -0.022 | -0.097, 0.055 | 0.115 | -0.010, 0.243 |
| **within-day inertia x Neuroticism** | **0.052** | 0.009, 0.094 | 0.039 | -0.002, 0.078 |
| **overnight inertia x Neuroticism** | 0.011 | -0.053, 0.076 | -0.009 | -0.069, 0.051 |
| *Random Effects (standard deviations)* | | | | |
| **SD intercept id** | **1.391** | 1.269, 1.532 | **1.733** | 1.583, 1.903 |
| **SD intercept id wave** | **0.449** | 0.403, 0.499 | **0.430** | 0.382, 0.483 |
| **SD intercept id wave day** | **0.375** | 0.331, 0.414 | **0.421** | 0.368, 0.469 |
| **SD overnight** | **0.187** | 0.106, 0.253 | **0.489** | 0.422, 0.564 |
| **SD within-day inertia** | **0.164** | 0.143, 0.187 | **0.150** | 0.130, 0.172 |
| **SD overnight inertia** | **0.187** | 0.145, 0.233 | **0.161** | 0.126, 0.202 |
| Observations | 26762 | | 26745 | |
| Marginal R^2^ / Conditional R^2^ | 0.112 / 0.653 | | 0.123 / 0.676 | |

**Table S11**

*Model Results for Perceived Stress as a Moderator of Affective Inertia*

|  | **NA** | | **PA** | |
| --- | --- | --- | --- | --- |
| *Model Parameters* | *Estimate* | *95% CI* | *Estimate* | *95% CI* |
| **Intercept** | **2.203** | 2.021, 2.383 | **6.118** | 5.899, 6.340 |
| **overnight** | -0.004 | -0.052, 0.044 | **-0.195** | -0.276, -0.115 |
| **perceived stress** | **0.530** | 0.378, 0.678 | **-0.476** | -0.635, -0.328 |
| **within-day inertia** | **0.241** | 0.211, 0.272 | **0.308** | 0.278, 0.338 |
| **overnight inertia** | **0.164** | 0.123, 0.206 | **0.158** | 0.120, 0.196 |
| **overnight x perceived stress** | -0.061 | -0.143, 0.023 | 0.121 | -0.000, 0.244 |
| **within-day inertia x perceived stress** | **0.079** | 0.042, 0.115 | **0.072** | 0.038, 0.105 |
| **overnight inertia x perceived stress** | 0.024 | -0.042, 0.090 | **0.086** | 0.027, 0.146 |
| *Random Effects (standard deviations)* | | | | |
| **SD intercept id** | **1.412** | 1.286, 1.556 | **1.791** | 1.631, 1.967 |
| **SD intercept id wave** | **0.439** | 0.394, 0.490 | **0.414** | 0.367, 0.465 |
| **SD intercept id wave day** | **0.377** | 0.332, 0.417 | **0.421** | 0.367, 0.470 |
| **SD overnight** | **0.185** | 0.103, 0.251 | **0.488** | 0.419, 0.564 |
| **SD within-day inertia** | **0.162** | 0.140, 0.185 | **0.149** | 0.129, 0.172 |
| **SD overnight inertia** | **0.185** | 0.144, 0.231 | **0.163** | 0.126, 0.204 |
| Observations | 26558 | | 26668 | |
| Marginal R^2^ / Conditional R^2^ | 0.055 / 0.654 | | 0.058 / 0.677 | |

Note. PSS = perceived stress scale

**Table S12**

*Model Results for Rumination as a Moderator of Affective Inertia*

|  | **NA** | | **PA** | |
| --- | --- | --- | --- | --- |
| *Model Parameters* | *Estimate* | *95% CI* | *Estimate* | *95% CI* |
| **Intercept** | **2.211** | 2.024, 2.398 | **6.112** | 5.882, 6.340 |
| **overnight** | -0.007 | -0.055, 0.041 | **-0.193** | -0.273, -0.114 |
| **Rumination** | **0.268** | 0.154, 0.390 | **-0.207** | -0.324, -0.091 |
| **within-day inertia** | **0.245** | 0.214, 0.276 | **0.311** | 0.280, 0.340 |
| **overnight inertia** | **0.159** | 0.116, 0.202 | **0.149** | 0.111, 0.188 |
| **overnight x Rumination** | -0.006 | -0.063, 0.050 | 0.033 | -0.052, 0.120 |
| **within-day inertia x Rumination** | 0.017 | -0.010, 0.043 | 0.022 | -0.004, 0.047 |
| **overnight inertia x Rumination** | 0.042 | -0.002, 0.087 | **0.070** | 0.028, 0.111 |
| *Random Effects (standard deviations)* | | | | |
| **SD intercept id** | **1.456** | 1.326, 1.602 | **1.834** | 1.674, 2.021 |
| **SD intercept id wave** | **0.447** | 0.401, 0.498 | **0.414** | 0.367, 0.466 |
| **SD intercept id wave day** | **0.376** | 0.333, 0.416 | **0.415** | 0.360, 0.466 |
| **SD overnight** | **0.190** | 0.104, 0.256 | **0.483** | 0.415, 0.560 |
| **SD within-day inertia** | **0.163** | 0.143, 0.187 | **0.152** | 0.133, 0.175 |
| **SD overnight inertia** | **0.184** | 0.143, 0.230 | **0.158** | 0.122, 0.199 |
| Observations | 26558 | | 26541 | |
| Marginal R^2^ / Conditional R^2^ | 0.042 / 0.654 | | 0.049 / 0.677 | |

**R Packages used in data preparation, analyses, and illustration**

**bayesplot**
Gabry, J., & Mahr, T. (2025). *bayesplot: Plotting for Bayesian Models* (R package version 1.12.0). https://mc-stan.org/bayesplot/
Gabry, J., Simpson, D., Vehtari, A., Betancourt, M., & Gelman, A. (2019). Visualization in Bayesian workflow. *Journal of the Royal Statistical Society: Series A (Statistics in Society)*, 182(2), 389–402. https://doi.org/10.1111/rssa.12378

**bayestestR**
Makowski, D., Ben-Shachar, M. S., & Lüdecke, D. (2019). bayestestR: Describing Effects and their Uncertainty, Existence and Significance within the Bayesian Framework. *Journal of Open Source Software*, 4(40), 1541. https://doi.org/10.21105/joss.01541

**brms**
Bürkner, P.-C. (2017). brms: An R Package for Bayesian Multilevel Models Using Stan. *Journal of Statistical Software*, 80(1), 1–28. https://doi.org/10.18637/jss.v080.i01

**cowplot**
Wilke, C. O. (2024). *cowplot: Streamlined Plot Theme and Plot Annotations for 'ggplot2'*. https://wilkelab.org/cowplot/

**GGally**

Schloerke, B., Cook, D., Larmarange, J., Briatte, F., Marbach, M., Thoen, E., Elberg, A., & Crowley, J. (2024). *GGally: Extension to 'ggplot2'.* https://doi.org/10.32614/CRAN.package.GGally

**ggplot2**
Wickham, H. (2016). *ggplot2: Elegant Graphics for Data Analysis*. Springer-Verlag New York. ISBN 978-3-319-24277-4. https://ggplot2.tidyverse.org

**gtsummary**
Sjoberg, D. D., Whiting, K., Curry, M., Lavery, J. A., & Larmarange, J. (2021). Reproducible Summary Tables with the gtsummary Package. *The R Journal*, 13, 570–580. https://doi.org/10.32614/RJ-2021-053

**haven**
Wickham, H., Miller, E., & Smith, D. (2023). *haven: Import and Export 'SPSS', 'Stata' and 'SAS' Files* (R package version 2.5.4). https://haven.tidyverse.org

**Hmisc**
Harrell Jr, F. E. (2025). *Hmisc: Harrell Miscellaneous* (R package version 5.2-4). https://github.com/harrelfe/Hmisc

**lubridate**
Grolemund, G., & Wickham, H. (2011). Dates and Times Made Easy with lubridate. *Journal of Statistical Software*, 40(3), 1–25. https://www.jstatsoft.org/v40/i03/

**misty**
Yanagida, T. (2025). *misty: Miscellaneous Functions.* (R package version 0.7.1). https://CRAN.R-project.org/package=misty

**patchwork**
Pedersen, T. L. (2025). *patchwork: The Composer of Plots* (R package version 1.3.0.9000). https://github.com/thomasp85/patchwork

**rio**
Becker, J., Chan, C., Schoch, D., & Leeper, T. J. (2024). *rio: A Swiss-Army Knife for Data I/O.* https://doi.org/10.32614/CRAN.package.rio

**sjPlot**
Lüdecke, D. (2024). *sjPlot: Data Visualization for Statistics in Social Science* (R package version 2.8.17). https://CRAN.R-project.org/package=sjPlot

**tidyverse**
Wickham, H., Averick, M., Bryan, J., Chang, W., McGowan, L. D., François, R., Grolemund, G., Hayes, A., Henry, L., Hester, J., Kuhn, M., Pedersen, T. L., Miller, E., Bache, S. M., Müller, K., Ooms, J., Robinson, D., Seidel, D. P., Spinu, V., Takahashi, K., Vaughan, D., Wilke, C., Woo, K., & Yutani, H. (2019). Welcome to the tidyverse. *Journal of Open Source Software*, 4(43), 1686. https://doi.org/10.21105/joss.01686
